# Human immunodeficiency: Extragonadal comorbidities of infertility in women

**DOI:** 10.1002/iid3.327

**Published:** 2020-07-03

**Authors:** Nahid Lorzadeh, Nastaran Kazemirad, Yasaman Kazemirad

**Affiliations:** ^1^ Department of Obstetrics and Gynecology, Faculty of Medicine Lorestan University of Medical Sciences Khorramabad Iran; ^2^ Faculty of Medicine Tehran University of Medical Sciences Tehran Iran; ^3^ Faculty of Dentistry Biruni University Istanbul Turkey

**Keywords:** extragonadal comorbidities, infertile women, infertility

## Abstract

**Introduction:**

Infertility is mediated by several changes system‐wide. These changes are likely to cause other systems‐related pathologies, such as changes in systemic immune response, particularly inflammatory response can lead to cardiovascular diseases and breast cancer.

**Methods:**

These morbidities can exist immediately or years after the diagnosis of infertility. Therefore, understanding the mechanism is important to move toward therapeutic interventions.

**Results:**

Several extragonadal pathologies are reported due to infertility, as well as, how these might also contribute to reproductive disabilities. Detailed evidence are still not present that can give stronger result.

**Conclusion:**

This review highlights some of the most frequent comorbidities that are seen in infertile women, hence requiring a need for complete clinical screening and care, as well as diagnosis and treatment in early stages.

## Graphical abstract

Several extragonadal pathologies are reported due to infertility, as well as how these might also contribute to reproductive disabilities. Women presenting subfertility or infertility need to be screened for abovementioned disorder. Adaptions in lifestyle might subsidize these effects.

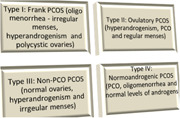

**Abbreviations**
ASA: anti‐sperm antibodies
cGMP: cyclic guanosine monophosphate
DHEAS: dehydroepiandrosterone sulfate
GLP: glucagon‐like peptide
IFN‐γ: interferon gamma
MCP: monocyte chemoattractant protein‐1
PCOS: polycystic ovary syndrome
PDE: cyclic nucleotide phosphodiesterase
SCF: stem cell factor
SM: sphingomyelin
TGF‐β: transforming growth factor‐β
TNF‐α: tumor necrosis factor‐α
VAT: visceral adipocyte tissues
VEGF: vascular endothelial growth factor

## 1 INTRODUCTION

Up to 18% of general population is prone to infertility.^1^ However, fewer studies target coexisting pathologies with infertility. Infertility is not a remote pathology and it can proffer effects on various systems in the body. Women infertility can have several causes, such as polycystic ovary syndrome (PCOS), endometriosis, tubal blockage, and hydrosalpinges.^2^, ^3^, ^4^ Despite, availability of diversity of treatment options, prevalence extragonadal pathologies might influence the treatment outcomes for infertility.^5^, ^6^

Certain dietary intake can also elevate the risk to attain infertility.^7^ A study reports genital infections like those in vagina, uterus, and ovaries that greatly contributes to infertility rather than cardiovascular factors.^8^

**Figure 1:**
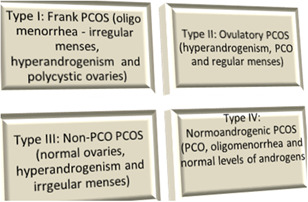
Various phenotypes of polycystic ovary syndrome

PCOS is characterized by various phenotypes (Figure 1) and thereby existence of comorbidities varies according to each category. In general, these women present elevated body mass index (BMI), follicle count and duration of menstrual cycles, hyperlipidemia, hyperandrogenism, insulin resistance, inflammation, and alterations in the morphology of ovaries.^9^

Similarly, endometriosis is an inflammatory disease, which is seen as the growth of endometrium tissues outside the uterus, where nearly half of such cases results in infertility. Several causes have been suggested, including housing of endometrial piece during menstruation near fallopian tube and immune dysfunction.^10^ Common symptoms are seen in the form of pelvic pain, absence of menses, increase in the levels of estrogens, and abnormal growth of endometrium. As the core of the disease, immune system plays a chief role in infertility, particularly due to inhibition of the activity of lymphocytes, natural killer (in peritoneal cavity) and cytotoxic T cell and aggravated macrophage and inflammatory response.^11^ Furthermore, the disturbance in production of cytokines, chemokines, and hormones has also found to worsen the disease.^12^ Abnormal production of uterine natural killer cells results in decrease in stem cell factor (SCF) in endometriosis, leading to infertility.^13^ Alteration in the Th‐1/Th‐2 skew, elevation of Th‐1 producing tumor necrosis factor‐alpha (TNF‐α), interleukin‐2 (IL‐2), and IL‐4 from Th‐2 promotes development of the pathology. This is mediated by the overexpression of T‐bet transcription factor leading to overproduction of Th‐1 and GATA3 for that of Th‐2, whereas decrease in the Foxp3‐caused Treg cells is inspected in endometriosis infertile women.^14^, ^15^ Nonetheless, in later phase of the development of endometriosis, endocrine and immune systems, together, are thought to play their role in the development of the disease.^16^

In addition, immune system is also chiefly involved in unexplained infertility. Treg cells are thought to play critical immunosuppressive role for fetus. Decrease in the levels of CD4^+^, CD25^+^, Foxp3 cells, transforming growth factor‐β (TGF‐β), lymphocyte adhesion, and chemotaxis are associated with idiopathic infertility. Additionally, anti‐sperm antibodies (ASA) can elicit immunity in both, men and women. Blood barrier safeguards exposure of immune cells with ASA in men, whereas, immunoregulatory mechanism of vagina and cervix play protective role in females. Sperm entering women with ASA are prone to be phagocytized. Also, presence of antibodies against antigen in seminal fluid also can lead to infertility.^17^

Other alterations are also reported in immune system due to chronic inflammation, as a result of infertility. This includes IL‐4 and ‐6, IFN‐γ (interferon gamma), and TNF‐α levels greater than control.^18^

This review is designed to highlight the studies and evidence present in regard to infertility and its extragonadal manifestations. Severity of the disease might call for the need of medical screening and subsequent treatment. We have discussed the systemic changes that can lead to prevalence of the diseases mentioned below.

## 2 CARDIOVASCULAR DISEASES, METABOLIC SYNDROME, AND DIABETES MELLITUS

Infertility in women is strongly linked with the development of metabolic syndrome (dyslipidemia, hypertension, insulin resistance, and obesity) and various cardiovascular abnormalities^15^. Even in women with infertility to certain extent and frequent miscarriages, the risk of cardiomyopathy is substantial.^19^

Numerous studies have shown incidence of cardiovascular diseases (CVDs) in infertile couples. They possess artherogenic characteristics such as increased serum cholesterol, triglycerides, C‐reactive protein (inflammation marker), reduction in high‐density lipoproteins, homocysteine, vascular endothelial growth factor (VEGF), and endothelial plasminogen activator inhibitor,^20^ which can be plausible outcomes of infertility‐mediated oxidative stress, increased levels of antimullerian hormone,^21^ or reduction in estrogen levels.^22^ To it, anomalies in menstrual cycles and infertility due to ovarian disorders^23^ also increase the risk of CVD, with or without hyperglycemia.^24^, ^25^ In a studies, it is also seen that cardiovascular disorders, mostly increased lipid prolife,^26^ adds to the risk to gestational diabetes, increased birth weight (above 4 kg, macrosomia), pre‐eclampsia and impairment in the release of bile from the liver. High‐density lipoprotein, on the other hand, provides health benefits against these.^27^, ^28^ Abnormalities in childhood lipid profiles are also related to prospective pregnancy complications and sterility.^28^, ^29^ Endometriosis, as a consequence of chronic inflammation, can lead to angina, coronary artery disease, hypertension, hypercholesterolemia, and myocardial infraction.^30^, ^31^ Abnormalities in endometrium are noted due to diabetes, obesity, hyperglycemia, and hyperlipidemia.^32^

PCOS patients also appear to have increased levels of palmitoyl sphingomyelin (SM), cyclic guanosine monophosphate (cGMP), and dehydroepiandrosterone sulfate (DHEAS).^33^ Elevated levels of sphingolipids are also one of the significant findings in CVD. They induce inflammatory response, plaque formation, valvular impairment, and ischemic heart disease.^34^ Nonetheless, contradictory outcomes are reported about association of DHEA with CVD.^35^ Up to 40% of PCOS patients present metabolic syndrome. These patients may present insulin resistance along with obesity.^19^ Cyclic nucleotide phosphodiesterase (PDE) is also involved in second messenger cGMP signaling. Deficiency of its isoforms, causing breakage of cell cycle, is reported to be linked with development of smooth muscles‐related CVDs such as atherosclerosis and development of metabolic syndrome. PDE3A (isoform) knockout mice, along with cardiovascular abnormalities also is deficit of matured oocytes and are barren.^36^

Flow‐mediated dilation (FMD), referring to the widening in the diameter of artery due to increased flow rate, is one of the significant markers of atherosclerosis due to endothelial dysfunction.^37^ Decrease in FMD in PCOS patients has been reported in several studies. Meta‐analysis concluded that more than 20% of these patients were at the risk of developing CVD due to a decrease in FMD. This might be due to visceral adiposity, oxidative stress, inflammatory response, and reduction of nitric oxide (NO) levels.^38^ Several evidence are presented in regard to the role of NO is PCOS. Krishna et al^39^ provided a detailed study concerning the role of NO and endothelial dysfunction in PCOS women. Owing to exacerbated inflammatory response and downregulated Treg immune action, studied revealed that decrease in NO levels is mediated by the downregulation of the enzyme involved in the suppression on the levels of l‐arginine (precursor of NO) by reducing the transcripts of the enzymes that arbitrate biochemical conversions and arginine transporter of arginine (cationic amino acid transporter). Additionally, increase in arginine degrader and asymmetric dimethylarginine (competitive inhibitor) also adds to this facilitates reduction in nitrite and nitrate (end products of NO). Conversely, treating PCOS women with clomiphene along with nitric oxide increases the chances of conceiving.^40^ However, contradictory animal models have been presented.^41^

Correspondingly, several studies have pointed out toward the concomitance of obesity, insulin resistance, and other metabolic disorders in all four phenotypes of PCOS.^26^ Hyperinsulinemia is characterized by excessive release of luteinizing hormone and obesity in polycystic ovary (PCO) patients independent of other factors.^42^ Insulin resistance is these patients is also depicted by the enhancement of inflammation (IL‐6 and TNF‐α), glycosylation end‐product leukocyte adhesion, and mitochondrial oxidative stress.^20^, ^43^ Additionally, mutations in the mitochondrial RNA is also associated severity of the symptoms.^44^ Treatment of oxidative stress leads to reduction in apoptosis and has therapeutic potencies in this area.^45^

Phenotype I is characterized by highest levels of fasting insulin, hypervolemic ovaries with greatest prevalence of cysts whereas, IV exhibits highest levels of luteinizing hormone (LH).^46^, ^47^ Bil et al^48^ showed in a study that phenotype I and II are at the greater risk to develop metabolic syndrome as compared with the other phenotypes, owing to the visceral adiposity index. Obesity in PCOS women is evident due to hyperandrogensim. It is established that cytokines released from adipocytes, adipokines particularly adiponectin, play significant role. Its expression inversely regulates adiposity, inflammation, lipid profile, and levels of glucose and insulin.^49^ Reduction in the levels of high molecular adiponectin is associated with cardiovascular disorder in type II diabetic patients.^50^ Recent study has shown that this isoform is also involved in the comorbidities of polycystic ovaries due to elevated sympathetic activity.^51^ Due to vast amount of evidence in relation with insulin resistance and hyperglycemia, studies have vividly shown the risk of diabetes embedded in infertile patients particularly due to tubal blockade and oligo or anovulation.^52^ Early animal studies have shown that expression of Cdk4 in beta islet of pancreas and pituitary gland is involved in anterior pituitary development, affecting secretion of prolactin. Complete absence of transcripts can lead to diabetes and infertility, concurrently.^53^

**Figure 2:**
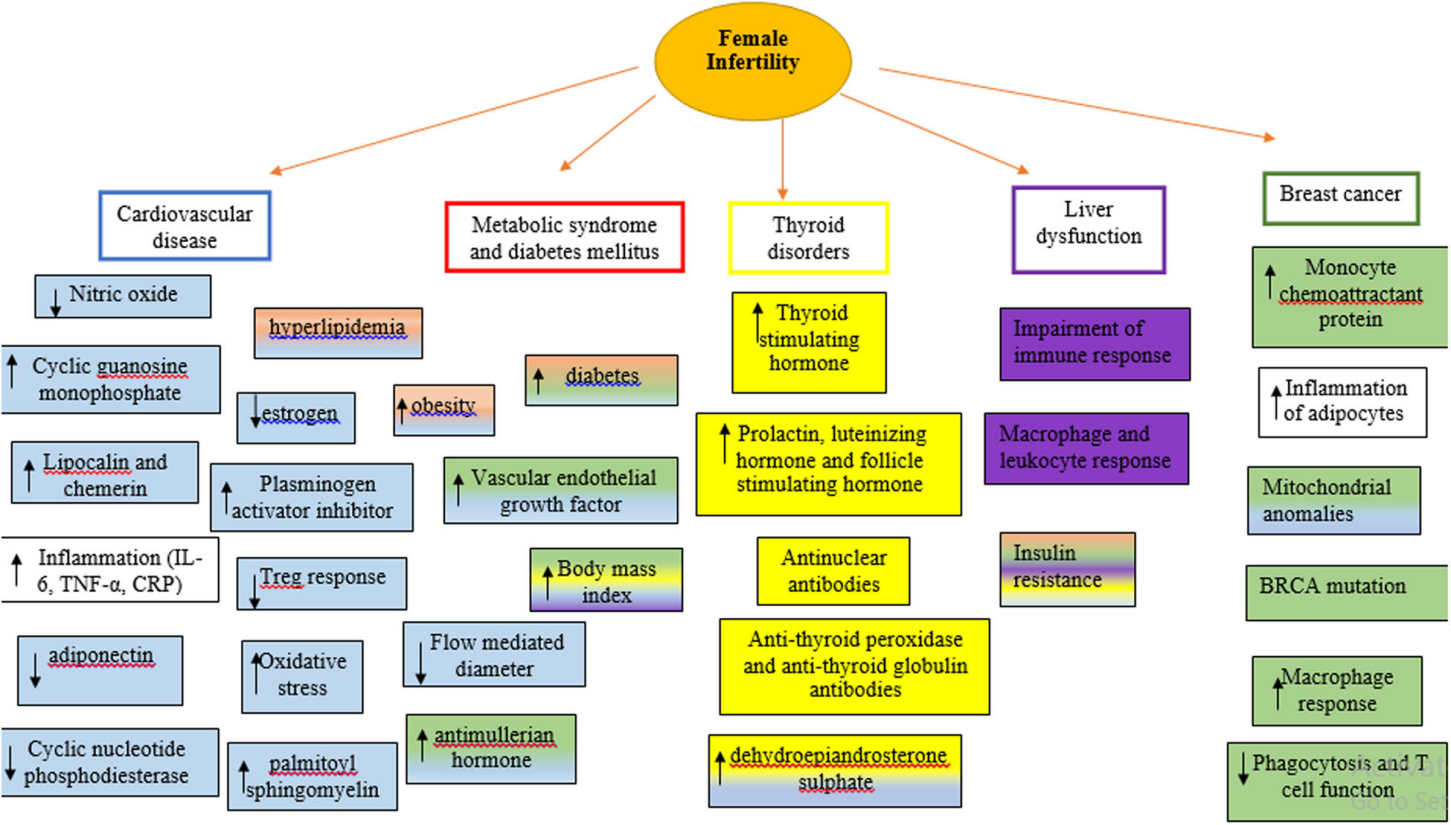
Effects of extragonadal comorbidities in relation to infertility. Figure shows changes that can lead that are seen in case of infertility and respective pathological condition (blue color: cardiovascular disease, red color: metabolic syndrome and diabetes, yellow color: thyroid dysfunction, purple color: liver disease, green color: breast cancer). BRCA, breast related cancer antigen; CRP, C‐reactive protein; IL‐6, interleukin 6; TNF‐α, tumor necrosis factor‐alpha

Additionally, raised levels of androgens in PCOS women are also seen by the reduction in the expression of adiponectin receptors. This is likely due to the impairment in the release of androgens by theca cells mediated by low levels of circulating adiponectin.^49^ Visceral adiposity in affected women is seen perceived by the increase in expression of chemerin and lipocalin‐2 (types of adipokines) in visceral adipocyte tissues (VAT), which is normally seen in men and opposite in healthy women^54^ (Figure 2).

## 3 THYROID DYSFUNCTION

Alteration in the levels of thyroid hormone is strongly related to female infertility. Hypothyroidism (elevated thyroid stimulating hormone, TSH) is also characterized by hyperprolactemia (increase in serum prolactin levels).^55^ Quintino‐Moroet al^56^ provided a study on Graves' disease (GD) and Hashimoto's thyroiditis (HT) and onset of infertility. It was found that approximately 50% of women presenting GD and HT are sterile, especially those aged greater than 35 years. Presence of antithyroid peroxidase and antithyroid globulin antibodies leads to the destruction of the gland. These autoimmune antibodies are likely to cause abortion and can chiefly contribute to infertility.^57^ Hypothyroidism is prevalent in infertile women and strongly affects the ability to conceive. Patients with hyperprolactemia and hypothyroidism responds significantly to thyroid hormone treatment and are able to conceive.^58^, ^59^ Increase in the production of prolactin and TSH can lead to excessive production of luteinizing and follicle‐stimulating hormone, DHEA, and accumulation of collagen in ovaries; hence, directing the formation of cysts in ovaries. Coexistence of chronic lymphocytic thyroiditis and PCOS and goiter and PCOS is also of greater prevalence than controls.^60^ Correlation between these pathological conditions can be comprehended by various evidence. Primarily, obesity and insulin resistance strongly associate with them. Insulin resistance suppresses deiodinase‐2 activity in pituitary gland, henceforth, decreasing T3, elevating TSH, and eliciting inflammatory response. Besides, obesity causes a rise in thyrotropin‐releasing hormone, advancing hypothyroidism.^60^ Presence of autoantibodies, antinuclear antibodies in PCOS patients, might as well cause autoimmune thyroid disease.^61^, ^62^

Women with adenomyosis are also at higher risk to develop thyroid cancer^63^ while on the other hand, hypothyroidism can lead to the endometrial cancer^64^ (Figure 2).

## 4 BREAST CANCER

Breast cancer is the second most recurrent type of cancer, dominant in women. Its association with infertility is studied widely with both, positive and negative, outcomes been strongly evident.

Breast cancer is associated with obesity and insulin resistance by immune‐system‐mediated hyperinsulinemia in adipocytes. Hyperproduction of macrophages instigates inflammatory response, as described above. M1 phenotype (inflammatory activity) is seen in the initial stage of tumorigenesis. Later macrophages switch to M2 phenotype—tumor‐associated macrophages (anti‐inflammatory and regulatory) and expresses VEGF, fibroblast growth factor, and monocyte chemoattractant protein‐1 (MCP). M1 also impedes action of insulin, leading to insulin resistance, diabetes mellitus, and obesity.^65^ In breast cancer, it inhibits phagocytosis and T‐cell function and promotes angiogenesis.^66^ To it, adipose tissue inflammation also corresponds to the inflammation in mammary glands.^67^ Contribution of estrogen is also noteworthy in breast cancer due to the activation of proinflammatory cytokines and adipokines.^68^ Adding to all these indications, it can be determined that infertility‐related pathogenesis such as PCOS, add significance to the incidence of breast cancer. However, studies have failed to find significant relationship.^69^, ^70^, ^71^ Assistive reproductive technology is also chiefly associated with the development of breast cancer due to induction of hormones.^72^ Women with mutations in BRAC (breast cancer) genes are seen to have decreased ovarian reserved and response rate as compared with controls, marked by the reduction in anti‐Müllerian hormone concentrations.^73^, ^74^, ^75^ Likewise, women with endometriosis also possess risk of acquiring breast cancer although, in this case as well, convincing evidence are not present.^76^ Studies have also depicted the interrelation between ovarian and breast cancer. BRCA1 mutation (ex9‐12del) is associated with 35% and 29% of ovarian and breast cancer, respectively.^77^ Mutations in these genes are also seen to cause miscarriages and decrease the chances of having children,^78^ due to its expression in germline cells and blastocysts. In addition, impairment of BRCA‐mediated DNA double‐stranded repair machine in oocytes can lead to the aging of eggs and alleviate follicular reserve^79^ (Figure 2).

## 5 LIVER DISEASE

Nonalcoholic fatty acid liver disease (NAFLD) includes several diseases such as fibrosis, cirrhosis, hepatic steatosis, and liver cancer and mostly coexist with insulin resistance, oxidative stress, mitochondrial anomalies, obesity, diabetes, and metabolic syndrome^80^. It can also lead to cardiovascular morbidities.^81^ Reduction in the levels of adipokines, instigation of local and systemic inflammatory response by adipocytes, macrophages, and leukocytes play critical role in the pathogenesis of NAFLD.^82^ Al‐Jaroudi et al^83^ studied the prevalence of NAFLD in PCO women and found that 60% of women presenting polycystic ovaries had liver disease with an increase in BMI and hyperlipidemia. Hyperandrogensim in PCOS is also correlated with NAFLD.^84^ Ovariectomy in endometrial cancer patients increases the risk of developing NAFLD, proportional to the increase in period between the operation and the development of the disease (up to 40% in 5 years of the surgery) along with the occurrence of diabetes and insulin resistance.^85^

Hepatitis B virus (HBV) leads to infertility directed by tubal and uterine causes. Impaired immune response is supposedly one of the noteworthy linkage between the two.^86^ Hepatitis can also affect the success of in vitro fertilization (IVF).^87^, ^88^

Glucagon‐like peptide (GLP) regulates secretion of insulin and maintains the balance between the requirement of sugar in the body by causing excess to be converted into glycogen. Its deficiency can lead to diabetes mellitus (type II) and recent case report has stated that infertile PCOS women when treated with GLP showed improvement in insulin resistance and ovarian function, with successful pregnancy.^89^ It also enhances hypothalamus‐pituitary‐mediated gonadal functions by increasing the secretion of luteinizing hormone.^90^ Similarly, lipin 1, expressed in liver, regulates the levels of estrogen. Animal models have shown that reduction in the levels of lipin is seen in diabetic mice with impaired fertility and elevated serum estrogen levels^91^ (Figure 2).

## 6 PSYCHOLOGICAL STRESS

Inability to conceive can impose psychological changes in sterile people.

Recent systematic review concluded that infertile women has greater prevalence of psychiatric disorders than controls.^1^ These women show a decrease in the ability to cope with the stress (resilience).^92^

Estrogen is widely studied for its role in temperamental changes. It is evident that it plays a significant role in regulation of mood and acts as antidepressants.^93^ Women perceiving assisted reproductive technology (ART) as the source to conceive child also present pretreatment depression/major depressive disorder, which impacts the success of the procedure.^94^, ^95^ Up to 60% of sterile women present depression, obsession, paranoia, and anxiety, especially those at younger age and in women.^96^, ^97^, ^98^ To it, miscarriages can also cause posttraumatic stress disorder. Psychological consultation is likely to improve chances of being pregnant and improve mental well‐being of infertile patients.^99^ Mind and body program, writing, and mindfulness are also effective in reducing this stress, thereby increasing the rate of pregnancy through IVF.^100^, ^101^, ^102^

**Figure 3:**
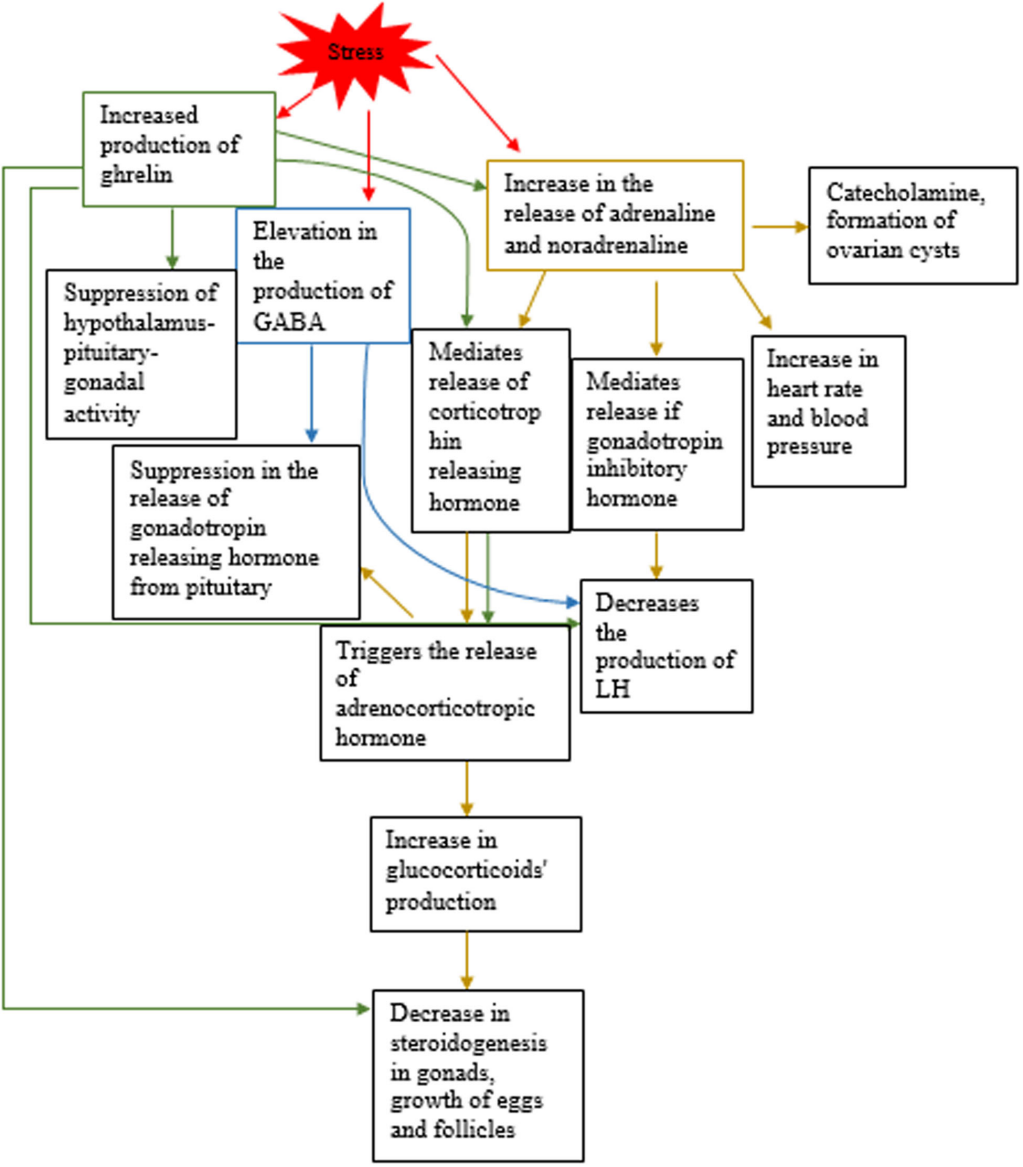
Stress induces the production of ghrelin (green), GABA (blue), and adrenaline and noradrenaline (yellow). The figure shows how they pose effects on reproductive system and have potencies to cause reproductive dysfunction. GABA, gamma aminobutyric acid; LH, luteinizing hormone

Interestingly, stress can also induce reproductive dysfunction. Individual presented with long‐term stress are at greater incidence to face infertility‐related outcomes as compared with controls. In addition, people suffering from infertility, seeking ART for fertilization also undergo financial and emotional anxiety. It results in overall decline in the success rate of IVF. Stress can lead to reduction in fertility by mediating the release of gonadotrophin‐inhibiting hormone, increasing the activity of sympathetic, and non‐sympathetic system leading to the release of corticotrophin‐releasing hormone and glucocorticoids, which can affect follicular growth, suppression of gonadotrophin‐releasing hormone by GABA (gamma aminobutyric acid) along with decline in kisspeptin expression. Besides, ghrelin (increased in response to psychological stress) also plays a significant role in the release of LH, ovarian development and steroidogenesis, and pregnancy.^103^ Figure 3 summarizes how stress poses effects on fertility.

## 7 CONCLUSION

Several extragonadal pathologies are reported due to infertility, as well as, how these might also contribute to reproductive disabilities. Detailed evidence are still not present that can give stronger result. However, women presenting subfertility or infertility need to be screened for abovementioned disorder. Adaptions in lifestyle might minimize these effects. Additionally, treatment of these disease might aid betterment in ART results and cause pregnancy. Similarly, treating grounds of infertility, such as PCOS, might also diminish presentation of these morbidities.

## CONFLICTS OF INTEREST

All the fees provided by research center fund and deployed accordingly.

## AUTHOR CONTRIBUTIONS

NL: conceptualized and designed the study, drafted the initial manuscript, and reviewed and revised the manuscript. NK: designed the data collection instruments, collected data, carried out the initial analyses, and reviewed and revised the manuscript. YK: coordinated and supervised data collection, and critically reviewed the manuscript for important intellectual content. All authors approved the final manuscript as submitted and agree to be accountable for all aspects of the work.

## ETHICS STATEMENT

All procedures performed in this study involving human participants were in accordance with the ethical standards of the institutional and/or national research committee and with the 1964 Helsinki Declaration and its later amendments or comparable ethical standards.
